# Frequent Countermeasure Usage by Narcissistic Examinees in the Concealed Information Test

**DOI:** 10.3389/fpsyg.2019.01068

**Published:** 2019-05-10

**Authors:** Eitan Elaad, Liza Zvi

**Affiliations:** ^1^Department of Behavioral Sciences and Psychology, Ariel University, Ariel, Israel; ^2^Department of Criminology, Ariel University, Ariel, Israel

**Keywords:** Concealed Information Test, countermeasures, narcissism, self-assessed lie-telling ability, self-assessed truth-telling ability, polygraph

## Abstract

Narcissistic dimensions and self-assessed lie and truth-telling and detecting abilities were used to predict deliberate attempts to influence the outcomes of the Concealed Information polygraph Test. In this study, which used a fabricated murder scenario, 241 examinees were randomly allocated to four experimental conditions in a 2 × 2 factorial design. Two guilt conditions (guilty and innocent) were crossed with two countermeasures conditions (with or without countermeasure instructions). One group consisted of 120 informed guilty participants who were offered the opportunity to give a false response to neutral items by verbally answering “yes,” by which they falsely confirmed that the item is relevant to the murder case. Participants were told that frequent lying would confuse the polygraph and help them pass the test. Another informed guilty group (41 participants) was not given the opportunity to use countermeasures. Two control groups of 40 participants each were unaware of the critical items. One control group used countermeasures while the other did not. Narcissistic dimensions and self-assessed lie-telling ability correlated positively with frequent use of countermeasures. Conflicting results about the relation between countermeasure usage and physiological responses to critical items were obtained.

## Introduction

### The Concealed Information Test

Extensive psychophysiological research on the Concealed Information Test (CIT) in laboratory settings indicates that the CIT is effective in detecting crime-related memories that knowledgeable examinees are trying to hide (Ben-Shakhar and Elaad, 2003; Meijer et al., 2014). In the CIT, participants are presented with a series of items, one of which is related to the investigated crime, and the others are irrelevant and serve as controls. The assumption is that the guilty suspect has a memory of the critical item and will therefore recognize it among the alternatives. Such focused attention on the critical item elicits enhanced physiological responses. Innocent examinees, without the relevant knowledge, are unable to distinguish between the critical item and controls. They are, therefore, less responsive to the critical items (Lykken, 1998) and are expected to respond unsystematically to the different items. Meijer et al. (2014) performed a meta-analysis on the validity of the CIT in laboratory studies and showed a large mean effect size (*d*) of 1.55 for skin conductance response amplitude, 1.11 for respiration line length, and 0.89 for heart rate. Hence, all three measures are valid detectors of concealed information.

### Countermeasure Effects

However, detection efficiency of the CIT might be compromised by countermeasures (CMs) which are deliberate actions examinees may use to influence their physiological responses during the test. CMs may appear in many forms. They may be physical (e.g., biting one’s tongue); they may be mental (e.g., counting backward by seven); they may be verbal (e.g., intentionally delaying the verbal answer); and they may be behavioral such as deliberately damaging the polygraph equipment (e.g., cutting the respiration tubes) or smearing glue on the finger tips. Finally, examinees may use drugs or alcohol (e.g., Bradley and Ainsworth, 1984; Iacono et al., 1984, 1992) as CMs in the CIT.

Earlier research indicated that using CMs may impair the validity of the CIT. The first published study (Lykken, 1960) indicated no CM effect on electrodermal responses. Another early study (Elaad and Ben-Shakhar, 1991) reported increased electrodermal detection when CMs were directed to the critical items and decreased detection when CMs were applied during the entire test. Honts et al. (1996), compared the effects of mental and physical CMs on the electrodermal and respiration measures. Participants were instructed to manipulate their responses to two predefined neutral items. Results indicated that the concealed information held by the typical guilty group (without CM manipulation) was better detected with skin resistance responses than that of the two CM groups. No such effect was obtained for respiration. In general, physical CMs were found to be more effective than mental CMs. Ben-Shakhar and Dolev (1996) used mental CMs and reported differences between two CM conditions and the typical guilty condition for electrodermal responses. Similar outcomes were not observed for respiration. Elaad and Ben-Shakhar (2009) reported that physical CMs reduced electrodermal efficiency but had a relatively small effect on finger pulse and respiration measures. On the other hand, electrodermal responses showed resistance to mental CMs. More recently, Peth et al. (2016), reported that electrodermal responses were more susceptible to CM usage than heart rate deceleration and respiration. Ocular measures (eye blink rate, fixation rate and duration) were more strongly affected by CMs than autonomic responses. Still, in all these studies, guilty participants (with and without CMs) showed stronger responses to critical items than did uniformed innocents.

In the studies cited above, participants received specific instructions on applying CMs to specific neutral items and were denied the freedom to choose for themselves whether, when, and how frequently to apply CMs. Such a procedure may have compromised the ecological validity of these studies. Furthermore, these studies failed to indicate the extent of manipulated neutral details in each study. The current study addresses these concerns. Participants were free to apply as many CMs to neutral items as they wished, and the number of CM attempts was accurately identified and recorded. Another group of guilty participants received no CM instructions and served as a control group, whose results were used to examine the effectiveness of the CMs. It was expected that both groups (with and without CM usage) would show increased physiological responding to critical items compared to neutral items, for all three measures: electrodermal, respiration, and cardiovascular responses. Based on previous results, CM usage was expected to reduce physiological detection of all three measures. Two additional control groups of uninformed innocent participants were used: one control group, to assess the frequency of CM use in the absence of any motivation to conceal information; and a standard control group with no-CMs, which provides a base rate for exonerating innocent examinees.

### The Lie-Truth Ability Assessment Scale

The current study was further designed to examine individual differences between participants’ use of CMs. To the best of our knowledge, this is the first effort to characterize participants who tend, more than others, to use CMs in the CIT. To this end, several scales were used. The lie-truth ability assessment scale (LTAAS) (Zvi and Elaad, 2018) was developed from previously used single questions (e.g., Elaad, 2009, 2015) to learn how people self-assess their ability to tell lies successfully (e.g., *in comparison with other people, how would you rate your ability to tell lies?*); tell the truth convincingly (e.g., *in comparison with other people, how good is your ability to convince others to believe your truthful statements when you are in trouble?*; detect lies efficiently (e.g., *in comparison with other people, how would you rate your ability to detect lies?*); and believe truths told by other people (e.g., *in comparison with your close acquaintances, how able are you to discern truthful statements?*).

A recent meta-analysis (Elaad, 2019) indicated that truth-telling ability is overestimated whereas lie-telling ability is not. The “truth-telling bias” – the excessive confidence people express in their ability to persuade receivers of their truthful communications – rests on the belief that telling the truth is a simple thing (Buller and Burgoon, 1996). This confidence also fits general human expectations that most communications are truthful and there is no reason for others to doubt our own truthful messages.

The truth-telling bias may also be clarified by the “illusion of transparency” (Gilovich et al., 1998). The illusion suggests that in communications, senders are anchored to their own internal feelings. According to the anchoring and adjustment heuristic (Tversky and Kahneman, 1974), senders realize that receivers are not exposed to similar information and try to adjust accordingly. However, their adjustments are insufficient, and they continue to believe that receivers will discern their internal states and trust them when they tell the truth. Finally, the high self-assessed ability to convince when telling the truth assists the human desire to sustain one’s positive self-image (Kaplar and Gordon, 2004). In contrast, lie-telling abilities are not similarly overestimated (Elaad, 2019). The belief that lie-telling is a difficult task may partly explain such findings. Lie-telling is difficult because the liar must create a new and never-experienced account. Although some lies are easily constructed when they are based on scripts of familiar experiences, examples of problematic lies are more available than easily expressed lies. The desire to maintain a positive self-esteem may also explain the findings. Thus, if I lack the ability to tell lies persuasively, I am eligible to believe that I am an honest person. Finally, the illusion of transparency (Gilovich et al., 1998) may provide another explanation. People mistakenly believe that their lies shine through (Vrij, 2008). Although senders realize that others are unable to perceive their inner-feelings as they do, their adjustment is insufficient, and senders fear that ultimately receivers can detect their lies.

The overestimated lie-detection ability (Elaad, 2018) has been explained by daily life experiences where people often meet truthful statements rather than deceptive ones. By believing statements made by others, people feel they are correct most of the time. Most deceptive messages remain undetected and receivers have no feedback about their lie-detection errors. In the absence of corrective feedback, receivers feel that they are able lie-detectors. This bias may be further explained by people’s tendency to think of themselves favorably. Norms rule that people should not allow themselves to be simply deceived. In support of this attitude, people would like to believe that their success in detecting lies is above average.

Finally, people tend to believe other people and overestimate their ability to believe truths other people tell (e.g., Elaad, 2011). Calling a truth-teller “a liar” is a serious accusation that raises feelings of guilt and often terminates the communication. By being able to believe others, people feel capable of avoiding such social obstacles.

The LTAAS (Zvi and Elaad, 2018) consists of 16 questions divided into four subscales describing the four assessed lie-truth related abilities. Answers were given on a scale ranging from 0 (*much worse than others*) to 100 (*much better than others*), with 50 (*as good as others*) serving as the midpoint. It was expected that the truth-telling, truth-believing, and lie-detection abilities would be overestimated, whereas the ability to tell-lies convincingly would not be overestimated.

It was hypothesized that people who score high on the lie-telling ability scale will use CMs during the CIT more frequently, compared with low scorers. The logic is that people who have confidence in their lie-telling ability will use lies more often, including in the CIT, compared with people who are less confident of their lie-telling ability. This hypothesis is examined in the current study. Further, it was hypothesized that a sense of purpose would predict frequent CM usage by high lie-telling ability scorers. In the absence of purpose, lie-telling ability will not correlate with the CM rate. The other three self-assessed abilities will not correlate with frequent CM usage.

### Narcissistic Personality Inventory (NPI)

Another trait that may predict frequent CM use in the CIT is narcissism. It has been previously demonstrated that narcissism and deceiving others are positively correlated (Oliveira and Levine, 2008; Giammarco et al., 2013; Baughman et al., 2014; Azizli et al., 2016). Oliveira and Levine (2008) found a positive correlation between narcissism and positive attitudes toward deceptive communication (lie acceptability). Giammarco et al. (2013) reported that narcissistic individuals believe that they are better liars than the average person. Finally, a linkage between narcissism and unethical behavior was demonstrated in everyday life situations (Baughman et al., 2014; Jonason et al., 2014; Azizli et al., 2016). Therefore, the link between narcissism and frequent CM use in the CIT seems very likely. This association is particularly relevant since narcissism is believed to be a predictor of violence and aggression and narcissists are overrepresented among criminals (Bushman and Baumeister, 2002; Larson et al., 2015). Nevertheless, no effort to demonstrate such a link has ever been made. When narcissism was studied as part of the Dark triad (Narcissism, psychopathy, and Machiavellianism), no association with the ability to deceive others or to detect lies in others was found (e.g., Wright et al., 2015; Wissing and Reinhard, 2017).

To measure narcissism, we used the 40-item Narcissistic Personality Inventory (NPI; Raskin and Hall, 1979; Raskin and Terry, 1988). Research indicates that the NPI can be divided into three subscales comprising 25 items from the original questionnaire: Leadership/Authority – capturing feelings of superiority and desire for power which is generally linked to adaptive outcomes; Grandiose Exhibitionism – capturing vanity and exhibitionism; and Entitlement/Exploitativeness – capturing entitled beliefs and exploitative behaviors (Ackerman et al., 2011). It was hypothesized that a higher overall narcissistic score, as well as higher scores of the three narcissistic components, predict increased use of CMs in the CIT.

### The Verbal “Yes” Response as a CM

The “yes” response was studied before in the context of the CIT (e.g., Kugelmass et al., 1967; Horneman and O’Gorman, 1985; Elaad and Ben-Shakhar, 1989). The purpose was to learn how the verbal response affects psychophysiological detection of critical items. We used the overt “yes” response as a CM. Unlike physical or mental CMs which are usually covert (i.e., the participants hide their use from the experimenter), answering “yes” to a control item is overt and immediately signals CM use to the experimenter. Still, in the present context, the “yes” manipulation conforms with the CM definition, namely, CMs are deliberate actions that are intended to influence the physiological responses during the CIT. The trade of ecological validity for the sake of precision in identifying and recording CMs coincides with the broader tendency in experimental studies that depart from actual situations in many respects in order to create a controlled situation in which processes and activities can be isolated from other factors and be accurately examined.

## Materials and Methods

### Participants

Two hundred and forty-one undergraduate Israeli students (207 females) enrolled in an introductory psychology program participated in the study for course credit. Their mean age was 22.6 (SD = 2.4). Based on self-reports 30.8% of the participants asserted that they were secular, 15.8% traditional (i.e., practice more religious rituals than secular participants), and 52.7% religious (one participant gave no answer). Participants read and signed a consent form that assured their confidentiality and anonymity and indicated that they were entitled to terminate their participation in the study at any time. All participants received course credit for participation and additional course credit for passing the polygraph test.

### Design

The study used a fabricated murder scenario presented to participants who were randomly assigned to four experimental conditions in a 2 × 2 factorial design. Two guilt conditions (informed guilty and uninformed innocents) were crossed with two CM conditions (with or without instructions to use CM). A group of 120 guilty participants were assigned to the role of the murderer and were accused of committing the crime. They were given the opportunity to use CMs by responding “yes” to control items during the CIT. Unlike earlier studies (e.g., Honts et al., 1996), participants were not restricted to a certain number of CMs. Another group of 41 guilty participants received no CM instructions and were instructed to respond “no” to all items (controlled for the CM effect). A third group of 40 uninformed innocent participants received CM instructions but as the CM served no clear purpose (they had no incriminating information to conceal), this group served to control for a possible purpose (motivation) effect. Finally, a group of 40 innocent participants served as a classical control group (uninformed innocents with no CM instructions). The three control conditions were designed to indicate group differences in physiological detection. According to the meta-analysis (Meijer et al., 2014) results, a large physiological effect size (at least *d* = 0.89), is assumed. We used a GPower analysis (Faul et al., 2009), to calculate the necessary sample size. For this end we used *F*-tests and a MANOVA with three repeated measures. The power level was 0.95 and α = 0.05. A lower sample size bound of *N* = 16 was computed. Hence, a sample size of 40 participants in each control condition is satisfactory. The fourth condition was designed to indicate individual differences and correlated narcissistic features, lying ability assessments and countermeasure frequency. In line with the results of Zvi and Elaad (2018), an anticipated small to medium effect size (*f*^2^ = 0.11) is assumed. Based on a power level of 0.95 and α = 0.05, an exact test for bivariate normal model correlation produced a lower sample size bound of *N* = 118.

### Data Acquisition: Physiological Responses in the CIT

Three physiological measures were used to examine the CIT process: (a) amplitude of the skin conductance response (SCR); (b) respiration line length (RLL); (c) finger pulse waveform length (FPWL). FPWL is not often used in experimental settings, and heart rate (HR) is preferred instead (e.g., Klein-Selle et al., 2017). Nevertheless, from an applied standpoint FPWL better reflects field practice than HR. All the mentioned indices have been previously used in similar studies and have proven to be valid markers of the changes that the CIT measures (e.g., Elaad, 2010, 2013).

Skin conductance was measured by a constant voltage system (0.5 V Atlas Researchers Ltd., Israel). Two Ag/AgCl Grass electrodes (0.8 cm diameter) were attached to the index and fourth fingers of the participants’ left-hand using contact jelly.

Respiration line length responses were recorded by an Atlas Researchers piezoelectric belt positioned around the thoracic area. Two additional covert respiration measures were recorded by respiratory piezoelectric effort transducers (Atlas Researchers) concealed in the back support of the polygraph examination chair and in the seat (Elaad and Ben-Shakhar, 2008). Elaad and Ben-Shakhar (2008) reported that the covert back respiration measure elicited responses similar to those elicited by the standard belt measure. As the recordings of the standard respiration measure are often affected by overlapping HR responses, it was decided to replace them with the more accurate covert back recordings. Such a replacement was used in previous studies (e.g., Elaad and Sommerfeld, 2016). From here on, the term RLL refers to covert respiration back recordings.

Finger pulse waveform length (FPWL) responses (Elaad and Ben-Shakhar, 2006) were recorded using an Atlas Researches (Israel) piezoelectric plethysmograph positioned around the right thumb. The plethysmograph measures pressure changes accompanying the blood volume pulse. An increase in these values represents vasodilation, whereas a decrease reflects vasoconstriction. FPWL also entails pulse rate changes.

### Apparatus

Since the apparatus of the present study was identical to that described in previous accounts (e.g., Elaad, 2013), we are repeating that description here: The experiment was conducted in an air-conditioned laboratory and monitored from a control room separated from the laboratory by a one-way mirror. A serial communication link from DAS (Data Acquisition System) was split in parallel into the serial ports of two PC computers. One computer controlled the stimulus presentation and computed skin conductance, respiration, and cardiovascular changes. The stimuli were displayed on a 19″ color monitor positioned in front of the participant. The second computer displayed physiological responses in real time in the form of graphs on a 19″ color monitor positioned in front of the experimenter located in the control room. The graphs were recorded for subsequent visual analysis and artifact control.

### Procedure

The ethics committee of Ariel University approved the present experiment. Two experimenters who performed two different roles (i.e., role-switching) conducted the experiment. Experimenter A welcomed the participants individually and informed them about the upcoming lie-detection study. Experimenter A explained that they would undergo a polygraph test about a hypothetical murder case. Participants further received a consent form to read and sign. By signing the consent form they indicated their agreement to participate in the experiment.

Participants completed a brief background questionnaire (name, gender, age, and level of religiosity), and were told that they would be granted four course credits: two for participation in the experiment and two as a bonus for passing the polygraph test. If they failed the polygraph test, they would lose the bonus.

Participants were next asked to self-assess their own lie-detection, lie-telling, truth-telling, and truth-believing abilities relative to other people. Assessments were made on the LTAAS (Zvi and Elaad, 2018). Participants then completed the NPI.

In the next stage of the procedure, participants were asked to select one of six closed envelopes positioned on a small table, open it, and read the instruction sheet inside which contained a description of a fabricated murder scenario.

### Guilt Manipulation

By selecting an envelope, participants in the guilt conditions assigned themselves to one of four crime profiles. Each profile specified six features of the fabricated murder case. The profiles were: (a) A murder took place in the “*David the Fisherman*” restaurant where Maurice Cohen was shot. The murderer, who was wearing a *red sweatshirt*, went in at *9 AM* shouted “*I am shooting*,” fired *three shots* at the victim and ran out leaving behind a *Baseball cap hat*; (b) A murder took place in the “*Chef in the Square*” restaurant where Maurice Cohen was shot. The murderer, who was wearing a *green shirt*, went in at *1 PM*, shouted: “*All on the floor*,” fired *four shots* at the victim and ran out leaving behind a *Beret hat*; (c) A murder took place in the “*Steak House*” restaurant where Maurice Cohen was shot. The murderer, who was wearing a *black jacket*, went in at *6 PM*, shouted: “*I will kill you*,” fired *two shots* at the victim and ran out leaving behind a *Casket hat*; and (d) A murder took place in the “*Herzl Skewers*” restaurant where Maurice Cohen was shot. The murderer, who was wearing a *white vest*, went in at *noon*, shouted: “*You deserve it*,” fired *one shot* at the victim and ran out leaving behind a *straw hat* (the critical items are marked in italics).

All profiles were followed by an identical text: “You are the murderer! The police suspect that you are aware of some details of the crime that only the murderer knows. In the following polygraph test, you will be asked about these details. If the polygraph detects at least two of the six critical details, you will lose the bonus of two credits that you now possess. If you successfully conceal your knowledge of at least five items from the polygraph, you will receive the bonus.” Guilty participants were asked to write down the six critical items on a separate sheet, place all the pages back into the envelope, and return to the experimenter.

### Innocence Manipulation

Participants in the innocent conditions picked an envelope describing the murder case in general terms as follows:

“A murder took place in a *certain restaurant* in Tel-Aviv where Maurice Cohen was shot. Witnesses at site indicated *the time* of the murder and told that the culprit *shouted something* before he *shot* the victim. Witnesses also described *what the murderer wore*, and the *hat* he left behind while leaving the scene of the crime.”

“You are innocent! Although innocent, the police suspect that you are aware of some details of the crime that only the murderer knows. In the following polygraph test, you will be asked about these details. If the polygraph determines that you are aware of at least two of the six critical items, you will lose the bonus of two credits that you now possess. If you successfully evade knowledge conclusions of at least five items by the polygraph, you will receive the bonus.” Innocent participants were instructed to place all the pages back into the envelope and return to the experimenter.

### CIT Explanation

All participants received the following explanation about how the polygraph test operates: “In the polygraph test, examinees are presented with a series of items, one of which is relevant to the interrogated crime and the others are irrelevant and serve as controls. Examinees are required to deny knowledge of the critical item by verbally responding “no” to each item. The “no” answer of innocent examinees, who have no critical knowledge, is a truthful statement for all presented items, critical and irrelevant alike. Guilty examinees, who recognize the critical items as part of the crime, are lying when denying knowledge of the critical items, and are truthful when responding “no” to control items. The polygraph is a lie-detector and can effectively detect lies of knowledgeable guilty examinees.”

### CM Manipulation

Guilty participants in the CM condition were informed that there is a method available that might help them pass the polygraph and retain their bonus credits despite their guilt. By responding “no” to critical items and “yes” to some (but not all) irrelevant control items, they would be falsely stating that the control items were part of the investigated crime. Consequently, the polygraph would be unable to differentiate between physiological responses accompanying lies to critical items and physiological responses elicited by lies to control items. Participants were told that they could use “yes” responses to control items whenever and how often they choose.

Participants were warned that this “multiple lie method” had not been previously tested, and that the polygraph might be able to distinguish between lies to the critical items (the knowledge of which they are trying to conceal) and lies to control items (designed to confuse the polygraph test). In such a case, the multiple lie method would not contribute to their success on the polygraph test.

Innocent participants in the CM condition received similar instructions but as they were not aware of the critical items no distinction was made between critical and control items. They were just told that answering “yes” to some items and “no” to the other items could help them pass the polygraph test and retain their bonus credits.

Participants in the “no-CM” conditions were not given these instructions.

### The Test Session

Experimenter A guided participants to the examination room where Experimenter B, who conducted the polygraph test was seated. Experimenter B was aware of the details of the experiment but was blind to the specific information that each examinee kept. Experimenter B invited the participants to sit in the examination chair, lean back into the back support, place their arms on the arm support, and avoid moving during the entire test. Experimenter B attached the polygraph sensors to the examinee and while doing so explained the function of each sensor (e.g., “the belt on your chest measures respiration”). Experimenter B told the examinees that they would take the polygraph test while they were alone in the examination room and would receive further instructions through a speaker. Experimenter B explained that the polygraph would determine whether they have knowledge about Maurice Cohen’s murder based on their physiological responses. Experimenter B then proceeded to the adjacent control room, closing the door of the examination room behind her/him.

An initial rest period of 2 min preceded the presentation of the questions. In that interval, skin conductance baseline was recorded. Six questions were presented, each focusing on a different feature of the murder case (the restaurant, the number of shots fired at the victim, the time of the murder, the clothes of the murderer, the words the murderer shouted, and the hat left behind).

The six questions were presented visually on a computer monitor and were read aloud to the examinees. Each question contained one critical and four neutral control items. The series of items was repeated twice. As control items, we used the items from the three unselected profiles and a fifth profile of neutral control items (*“Provence” restaurant, Brown suit, 8 PM*, “*Don’t move,” five shots, Kangol Bermuda hat*). One item from a sixth buffer profile of neutral items (*“Europa” restaurant, Blue suit, 10 AM*, “*here it comes,” six shots, Yarmulke*) was presented at the beginning of each question to absorb the initial orienting response.

The order of the five items within each question was random. Interstimulus intervals ranged from 16 to 24 s, with a mean interval of 20 s. In the no-CM condition, participants were instructed to respond verbally “no” to all item. Thus, guilty examinees lied when they answered “no” to the critical items and told the truth when they responded to the neutral items. In the CM condition, participants were given a choice to respond either “yes” or “no” and were not limited in the number of “yes” answers they gave. Here, guilty participants lied when they answered “no” to critical items and when they answered “yes” to neutral items. The questions were presented in random order, with a short break after three questions to allow participants a rest. Experimenter B recorded the examinees’ answers (“yes” or “no”) to each item within each question.

Immediately following the CIT, participants were detached from the polygraph and returned to Experimenter A, who asked them several questions about the test: (a) To what extent did they feel guilty or innocent? (b) How excited were they during the polygraph test? (c) How successful were they in concealing knowledge from the polygraph? and (d) How often did they apply CMs during the CIT (only the CM groups)? Finally, guilty participants were asked to name the items that they remembered from the description of the mock murder. Upon completion, participants were debriefed about the purpose of the study and informed about the bonus they earned.

## Results

### Manipulation Check

#### Guilt Manipulation

After the polygraph test, participants were asked to indicate to what extent they felt guilty or innocent in the mock murder case on a scale from 0 (*innocent*) to 100 (*guilty*). Results indicated that guilty participants rated themselves in the middle of the scale (Mean = 52.17, SD = 43.3). While they realized that their role was to simulate guilt, they found it difficult to identify with their role, a situation that is often observed in experimental mock crime studies. In comparison, innocent participants rated themselves low on the guilt scale (Mean 15.75, SD = 28.8). The difference between the groups was significant, *t*_(239)_ = 6.82, *p* < 0.001, *d* = 0.93, indicating that the guilt manipulation was effective.

#### Excitement

Participants were further asked to indicate their level of excitation during the test. Answers were given on a scale ranging from 0 (*not at all excited*) to 100 (*very excited*). No significant differences on the excitement scale scores between guilt CM, (Mean 54.5, SD = 26.7), guilt no-CM, (Mean = 52.7, SD = 25.6), and innocent no-CM (Mean = 52.9, SD = 27.0) conditions were found. The innocent CM group rated their excitement level lower (Mean = 41.8, SD = 25.4). The observed excitement scores indicate that participants were not very excited during the test, mainly because they realized that they were participating in an experiment.

#### Perceived Success

Participants were asked to assess their success in concealing the critical items from the polygraph on a scale ranging from 0 (*no success*) to 100 (*very successful*). Results showed that both guilty groups felt unsuccessful: The guilty CM group (Mean = 46.7, SD = 22.7) scored at a low level similar as that of the guilty no-CM group (Mean = 44.7, SD = 23.5). Innocent participants felt more successful. The rating of the innocent CM group (Mean = 59.3, SD = 24.2) was significantly higher than that of the guilty CM group, *t*_(158)_ = 2.99, *p* = 0.003, *d* = 0.54, and the score computed for the innocent no-CM group (Mean = 54.0, SD = 17.5) was significantly higher than that of the guilty no-CM group, *t*_(79)_ = 2.01, *p* = 0.046, *d* = 0.45. To conclude, innocent participants felt more successful than guilty participants. Employing CMs had no effect on participants’ perceived success in the test.

#### The Use of CMs

The CM groups were asked to indicate how often they answered “yes” to neutral items in the CIT. Answers were given on a scale ranging from 0 (*many more* “*no” responses than “yes” responses*) to 100 (*many more “yes” responses than “no” responses*). Answers correlated highly with the actual “yes” response rate to neutral items, *r*_(120)_ = 0.56, *p* < 0.001 and *r*_(40)_ = 0.72, *p* < 0.001, for the two guilty and innocent CM groups, respectively. Results indicated that participants were highly aware of their CM performance on the test.

### Memory of the Critical Items

Once the polygraph test was over, guilty participants were asked to indicate the items that they remembered from the mock murder case (mean = 5.62, SD = 1.01 items, out of 6). The obtained recall rate was high and resembled other recall rates obtained in previous CIT studies when a polygraph test was administered immediately after the execution of the mock-crime. For example, Elaad (2015) reported a similar recall rate (Mean = 5.63, SD = 0.68). Results show that there was no memory problem in the present study. Innocent participants were given the option to indicate that they were not aware of any critical item. All innocent participants marked this option.

### Statistics for Self-Assessed Lie and Truth Related Abilities and Narcissistic Dimension

The means and standard deviations of the self-assessed abilities to tell and detect lies and truths are presented in Table 1. Table 1 shows that participants overestimated their lie-detecting, truth-believing, and truth-telling abilities (the lower bound of the CI is larger than the midpoint – as good as others), whereas the lie-telling ability was not overestimated. Using matched samples *t*-tests, participants’ truth-telling ability was rated higher than either their lie-detecting ability, *t*_(240)_ = 7.17, *p* < 0.001, *d* = 0.54, or their truth-believing ability, *t*_(240)_ = 6.43, *p* < 0.001, *d* = 0.52. These results are in line with previous studies (e.g., Elaad, 2015).

**Table 1:** Percent means (and SDs) of self-assessed abilities to tell and detect lies and truths.

	Tell lies	Detect lies	Tell truths	Believe truths
Mean and SD	48 (17.5)	59 (14.6)	66 (12.4)	59 (13.8)
95% CI	46.0–50.5	56.8–60.6	64.3–67.4	57.2–60.8
Cronbach’s alpha	0.896	0.906	0.770	0.786


**N* = 241; CI, confidence interval in standard error units.*

Further, for the entire sample (*N* = 241), the Cronbach’s alpha computed for the 40 questions that comprise the total narcissistic score, was 0.93. Cronbach’s alphas were also computed for each subscale: 0.76, 0.82, and 0.49 for Leadership/Authority (11 items), Grandiose Exhibitionism (10 items), and Entitlement/Exploitativeness (4 items), respectively. The low reliability score for Entitlement has been observed before (Ackerman et al., 2011) and was explained by the small number of items that comprise the subscale.

### CM Scoring

The number of “yes” answers given to control items in the two CM conditions was summed for each participant and served as an index for the tendency to use CMs in the CIT. Findings show that guilty participants used significantly more CMs (Mean = 14.01, SD = 12.1) than innocent participants (Mean = 6.75, SD = 10.8), *t*_(158)_ = 3.35, *p* = 0.001, *d* = 0.62.

### Correlates of Narcissistic Features, Self-Assessed Lie- and Truth-Related Abilities and CM Use in the CIT

A positive correlation between self-assessed lie-telling ability and CM use by guilty participants (*N* = 120) was obtained (*r* = 0.21, *p* = 0.021, 95% CI = 0.08–0.33). Specifically, the higher the self-assessed lie-telling ability the greater the number of CMs they applied in the CIT. A regression analysis indicated that guilty participants’ lie-telling ability assessment explains 5% of the variance in the number of their “yes” answers to neutral items. Similar correlations with the CM frequency computed for the self-assessed lie-detecting (*r* = 0.15), truth-telling (*r* = 0.17), and believing (*r* = -0.14) abilities, were not significant.

The correlations between narcissistic dimensions and the frequency of CM attempts to neutral items are displayed in Table 2. Results indicate that for the 120 guilty participants, the total narcissistic score and all narcissistic subscale scores were positively correlated with frequent CM use. Specifically, guilty participants who scored high on all facets of narcissism tended, more than lower scorers, to apply CMs in the test. A regression analysis indicated that the total narcissistic score predicted 10% of the variance in CM use of guilty examinees.

**Table 2:** Correlations between narcissistic dimensions and CM frequencies computed for guilty participants in the CIT.

	Narcissism	Leadership/	Grandiose	Entitlement/
		Authority	Exhibitionism	Exploitativeness
Guilty *N* = 120	0.32^∗∗^	0.26^∗∗^	0.27^∗∗^	0.23^∗^
95% CI	0.15–0.47	0.09–0.42	0.09–0.42	0.05–0.39


*^∗^*p* < 0.05; ^∗∗^*p* < 0.01.*

Finally, it is useful to look at the correlations between assessments of the lie- and truth-related abilities and narcissistic feature for the entire sample (*N* = 241). Table 3 shows that lie-telling, lie-detecting, and truth-telling correlated positively with the overall narcissistic score. A more detailed inspection revealed that lie-telling was related to Grandiose Exhibitionism thinking. Lie-detection and truth-telling were related to all three narcissistic dimensions.

**Table 3:** Correlations between narcissistic dimensions and self-assessed lie-truth related abilities.

	Tell lies	Detect	Tell	Believe
		lies	truths	truths
Narcissism total score	0.21^∗∗^	0.24^∗∗^	0.36^∗∗^	0.11
95% CI	0.08–0.337	0.12–0.35	0.25–0.46	-0.02 to 0.23
Leadership/Authority	0.10	0.26^∗∗^	0.38^∗∗^	0.12
95% CI	-0.2 to 0.23.	0.10–0.37	0.27–0.49	-0.01 to 0.24
Grandiose Exhibitionism	0.35^∗∗^	0.30^∗∗^	0.38^∗∗^	0.06
95% CI	0.23–0.42.	0.18–0.41	0.28–0.49	-0.07 to 0.18
Entitlement/Exploitativeness	0.06	0.16^∗∗^	0.30^∗∗^	0.16^∗^
95% CI	-07 to 0.18	0.03–0.28.	0.18–0.41.	0.3–0.28


*^∗^*p* < 0.05, ^∗∗^*p* < 0.01, *N* = 241.*

### Analysis of the Physiological Measures

Individual differences in physiological responses dictate the use of within-subject standard scores relative to the respective means and standard deviations. All the responses to each CIT multiple choice questions were therefore transformed into standard scores. Essentially, standardization transformation was similar for all three measures, but since responses are indicated by smaller rather than larger RLLs and FPWLs, these *Z* scores were multiplied by -1.

For each measure, mean standardized responses to the critical alternatives were computed across all CIT series. These means served as the detection score of that participant. Because uninformed innocents were unaware of a critical profile, a critical profile was arbitrarily assigned to them. The mean standard scores computed for each physiological measure are presented separately for each of the four conditions, in Table 4. It is evident that for both guilt conditions, the *Z* scores of the three measures were significantly higher than chance (the lower bounds of the 95% confidence intervals are above 0) whereas the two innocent groups responded at chance level.

**Table 4:** Means (and SDs) of *Z* scores computed for the three physiological measures and the combined measure, in the four experimental conditions.

	SCR	FPWL	RLL	Combined
**Guilty CM (*N* = 120)**				
Mean and SD	0.304 (0.334)	0.264 (0.316)	0.261 (0.337)	0.276 (0.227)
95% CI	0.244–0.365	0.207–0.321	0.200–0.322	0.235–0.317
**Guilty no-CM (*N* = 41)**				
Mean and SD	0.446 (0.472)	0.402 (0.350)	0.284 (0.444)	0.384 (0.334)
95% CI	0.317–0.615	0.292–0.513	0.144–0.424	0.279–0.490
**Innocent CM (*N* = 40)**				
Mean and SD	-0.098 (0.618)	-0.008 (0.275)	-0.007 (0.241)	-0.038 (0.238)
95% CI	-0.296 to 0.100	-0.096 to 0.080	-0.084 to 0.070	-0.114 to 0.039
**Innocent no-CM (*N* = 40)**				
Mean and SD	-0.033 (0.232)	-0.006 (0.309)	0.032 (0.283)	-0.002 (0.177)
95% CI	-0.107 to 0.041	-0.105 to 0.093	-0.059 to 0.122	-0.059 to 0.054


*CI, confidence interval.*

Table 4 clearly shows that CMs had no effect on innocent participants’ physiological responses to critical items. Therefore, we further assessed CM effects by considering only the two guilty groups. A multivariate analysis of variance (MANOVA) was applied with CM instructions (CM, no-CM) as the independent factor and SCR, FPWL, and RLL as the dependent factors. A significant CM effect was obtained for SCR, *F*_(1,159)_ = 5.72, *p* = 0.018, $\eta_{p}^{2}$ = 0.04, and FPWL, *F*_(1,159)_ = 5.52, *p* = 0.020, $\eta_{p}^{2}$ = 0.03. RLL produced no significant CM effect, *F*_(1,159)_ = 0.12, *p* = 0.726. Results indicate that SCR and FPWL responses declined when CMs were applied. CM instructions did not affect RLL responses (Table 4). A combined physiological measure defined as (SCR+FPWL+RLL)/3 was also computed and is displayed in Table 4. A univariate analysis of variance (ANOVA), used on the combined measure showed similar significant results, *F*_(1,159)_ = 5.33, *p* = 0.022, $\eta_{p}^{2}$ = 0.03.

### Signal Detection Theory (ROC Analysis)

The effect sizes obtained for SCR and FPWL responses were rather low, which dictates caution when the significance levels of the results are considered. To support the CIT efficiency conclusions, a method derived from Signal Detection Theory, known as the Receiver Operating Characteristic (ROC) procedure, was employed. The ROC procedure has been used in previous CIT studies (e.g., Ben-Shakhar, 1977; Elaad and Ben-Shakhar, 1989, 1997, Vossel et al., 2003; Verschuere et al., 2007; Elaad, 2010; Zvi et al., 2012, 2015; Zvi and Elaad, 2016), and was recommended by the National Research Council Report (2003) as an appropriate method for describing the diagnostic value of polygraph tests. The ROC method defines detection efficacy as the degree of separation between the distributions of the responses to the critical items produced by the experimental and the control groups. The mean *Z* scores distributions computed for each participant across all the items of the critical profile were calculated for each physiological measure. ROC curves were then generated based on the distributions of the guilty groups (with and without CM instructions) and of the innocent control groups. As both innocent groups showed no differential responses to critical items, both were combined into a single control group of 80 participants.

Based on Bamber (1975), the areas under the ROC curves along with the corresponding 95% confidence intervals were computed for each guilt condition. The area statistic shows the detection efficacy of the tested measure across all possible cutoff points. The assumed values of the ROC area range between 0 and 1, so that an area of 1 indicates a perfect separation between the results obtained for the tested (guilty) and the control (innocent) distributions, whereas an area of 0.5 implies complete overlap of the results obtained by the two distributions. ROC statistics computed for each physiological measure (SCR, FPWL, RLL) are presented in Table 5. Table 5 indicates that the ROC area computed for all three measures was significantly larger than chance (the lower bounds of the ROC areas are no less than 0.5). This finding implies that both groups of guilty participants responded to the critical information at a significantly higher level than innocent participants.

**Table 5:** Areas under the ROC curves and related statistics computed for the three physiological measures and for the two CM conditions.

Measures	Area	Standard error	95% confidence interval
**No countermeasures used**			
SCR	0.808	0.043	0.723–0.893
FPWL	0.808	0.045	0.719–0.897
RLL	0.679	0.056	0.569–0.789
**Countermeasures used**			
SCR	0.778	0.033	0.714–0.843
FPWL	0.740	0.035	0.672–0.808
RLL	0.715	0.036	0.646–0.785


*No countermeasures used – N Negative = 80 N Positive = 41; countermeasures used – N Negative = 80 N Positive = 120.*

While these results were expected, the more interesting question is whether and to what extent did CM impair detection of the critical items. To explore this question, we compared the ROC areas computed for guilty participants with no-CM instructions with the ROC areas of guilty participants who applied CMs, using a method proposed by Hanley and McNeil (1983). None of the comparisons revealed a significant difference (*Z* = 1.19, *Z* = 0.55, and *Z* = -0.54, for the respective, SCR, FPWL and RLL measures). Results suggest that the present CMs were not effective in reducing detection of concealed information.

### Self-Assessed Lie- and Truth-Related Abilities, Narcissistic Dimensions, and Relative Physiological Responses to Critical Items

We correlated lie- and truth-related ability scores and the physiological responses (SCR, RLL, and FPWL) to critical items in the CIT elicited by guilty participants (*N* = 161). No significant correlation was found. Similarly, correlations between the total NPI score and the three subscale scores with the physiological responses to critical items, elicited by guilty participants, were computed. No significant correlation was found. Results appear in Table 6. It seems that self-assessed lie– and truth related abilities and narcissistic features have no relations to the responses of informed participants in the CIT.

**Table 6:** Correlations of relative physiological responses in the CIT with narcissism and its three dimensions and with self-assessed lie- and truth-related abilities.

	SCR	FPWL	RLL
Narcissism	0.027	0.065	0.029
Leadership/Authority	0.002	0.039	-0.025
Grandiose Exhibitionism	-0.008	0.047	-0.001
Entitlement/Exploitativeness	-0.010	0.042	0.025
Tell lies	-0.052	-0.003	-0.010
Detect lies	0.056	0.035	0.138
Tell truths	-0.031	0.062	-0.015
Believe truths	-0.142	0.014	-0.043
*N* = 161			


## Discussion

The purpose of the present study was to report the results of a preliminary exploration of attributes of people who tend to use frequent CMs in the CIT. To this end, we provided some participants with an opportunity to use CMs whenever they wished. Other participants were not exposed to CM instructions. As expected, guilty participants used CMs more frequently than uninformed innocent participants, indicating that a deliberate attempt to obstruct the test requires a purpose. It was further predicted that people who score high on narcissistic qualities and have high self-assessments of their lie-telling ability will more frequently use CMs than less narcissistic people and lower raters of their own lie-telling ability. Results supported our predictions for guilty participants.

Many people give low ratings to their ability to tell lies convincingly (Ekman and O’Sullivan, 1991; Elaad, 2003, 2011; Vrij, 2008). Such a bias has been observed among students (Elaad, 2011), prisoners (Elaad, 2009), laypersons (Elaad, 2009), adolescents (Elaad et al., 2012), and law enforcement personnel (Elaad, 2003). The desire to sustain a positive self-image contributes to the low lie-telling ability assessment. Thus, if one is not an able lie-teller, one is entitled to believe that one is an honest person. Nevertheless, the lie-telling ability is not always underestimated (see review by Elaad, 2018) since some people tend to rate their lie-telling ability rather high. For example, a group of police investigators (Elaad, 2009) and a group of male students (Zvi and Elaad, 2018) were found to give such ratings to their lie-telling abilities. Similar results were obtained in the present study. It seems that some people consider lying to be a positive quality that serves them well in social situations (Kashy and DePaulo, 1996). In summary, the two opposite tendencies toward lie-telling are active when lie-telling abilities are assessed.

The lie -and truth related ability assessments integrate into a more general concept of self-efficacy (Bandura, 1977). Bandura defined self-efficacy as people’s belief in their ability to accomplish goals in given situations. He described these beliefs as determinants of how people think, behave, and feel. In this context the observed variability in lie-telling ability assessments may promote interest in how people who rate high their lie-telling ability feel, think and behave in various social interactions.

Zvi and Elaad (2018) found that participants who assessed themselves as competent liars reported more lie-telling to a greater number of people than participants who assessed themselves as less competent liars. The association between lie-telling ability assessments and actual lying received further support in the present study where high lie-telling ability raters, who simulated the role of a guilty suspect, frequently used CMs in the CIT.

Narcissistic features have been found to be associated with deception (Oliveira and Levine, 2008; Giammarco et al., 2013; Baughman et al., 2014; Azizli et al., 2016). Specifically, Oliveira and Levine (2008) found that narcissism was associated with positive attitudes toward deceptive communication. Giammarco et al. (2013) reported that narcissistic individuals believe themselves to be better liars than the average person. Other accounts have linked narcissism to lying or unethical behavior in various everyday life situations (Baughman et al., 2014; Jonason et al., 2014; Azizli et al., 2016). It was therefore hypothesized that narcissism would predict more frequent CM use in the CIT (which are basically lies). The hypothesis was supported by the present results, given that frequent CMs served the purpose of hiding concealed information.

Three components of the NPI: Leadership/Authority, Grandiose Exhibitionism and Entitlement/Exploitativeness (Ackerman et al., 2011) proved to be good predictors of frequent CM use in the CIT. Leadership/Authority is linked with psychological health, adjustment, social potency, and other adaptive self-enhancement tendencies. This subscale captures confidence, assertiveness, and beliefs of leadership potential. It is positively correlated with self-esteem and a reduced propensity toward internalizing psychopathology. In sum, this dimension describes normal (in contrast to pathological) narcissism. High Leadership/Authority scorers have confidence in their ability to successfully apply CMs and beat the test. Grandiose Exhibitionism captures vanity and exhibitionism, the need to be the center of attention, to show off and be complimented. Participants who score high on the Grandiose Exhibitionism component apply CMs to receive compliments for their achievements in beating the test.

Entitlement/Exploitativeness seems to have more consistent and stronger associations with maladaptive outcomes. High Entitlement/Exploitativeness scorers possess lower self-esteem and exhibit lower levels of empathy and social desirability accompanied by lack of concern for others. Other features of high Entitlement/Exploitativeness scorers are increased mood variability and neuroticism. These participants use frequent CMs because they feel entitled to receive favorable results in the polygraph test. Using CMs is the best way to achieve a favorable result and be rewarded.

High lie-telling ability ratings correlated positively with the total NPI score and with the Grandiose/Exhibitionism subscale. Specifically, people with narcissistic qualities, who are obsessed with the need for compliments and approval, consider lie telling a legitimate means to obtain the admiration they seek. In this context, high ratings of their lie-telling ability serve their needs well. Lie-detection and truth-telling ability assessments correlated positively with the NPI total score and with all three dimensions. These results replicate and support the results of a recent study conducted on male students (Zvi and Elaad, 2018). As the present sample comprised mainly female students, the replication is important. It seems that people who have confidence in their social potency, and/or are driven by a desire to receive compliments and admiration of others, and/or feel entitled to special treatment, rate high their abilities to detect lies and to be convincing when telling the truth – two socially desirable qualities that people would like to include in their repertoire.

CM effects on physiological responses to critical items produced conflicting results. It seems that some CM effect exists, but it is weak. The “yes” answer is conspicuous and does not require much attentional effort. In contrast, common CMs (physical and mental alike) are inconspicuous and demand more attention from the examinee than the yes answer. It is therefore likely that these CMs are more effective in beating the polygraph test. The task of future research is to replicate the present study using more demanding CMs.

Other more general findings lend support to previous findings on the four lie-truth related abilities. It turned out that the truth-telling ability was rated higher than all other abilities, a result that has been consistently found in previous studies (e.g., Elaad, 2015). The dominance of the truth-telling ability assessment is consistent with the belief that telling the truth is a simple matter of “telling it like it is” (e.g., Buller and Burgoon, 1996) and that telling the truth is cognitively simpler than lying (Vrij et al., 2006; Gamer, 2011; Verschuere, 2016).

### Limitations

The CMs used in the present study are not usual CMs. Typically, CMs are applied to create artificial excitation to neutral items to prevent the polygraph operator from detecting examinees’ guilt (e.g., Ben-Shakhar and Dolev, 1996; Honts et al., 1996). In the present study, we used noticeable CMs in order to measure them accurately. It is the task of future research to reexamine our results with inconspicuous CMs.

Furthermore, narcissism and self-assessed lie-telling ability are only two of many possible personality and situational factors (e.g., stress situations, coping strategies, differences in stress appraisal, different values, professional expertise in lie-telling) that might influence the use of CMs in the CIT. Additional research is required to trace these factors.

The present sample of participants consisted mainly of young Israeli female students. In contrast, most real-life CIT examinees are male suspects from the general population with a diverse age and education distribution. Therefore, caution is dictated when the present results are considered. Future research is advised to replicate the present study on different samples (e.g., prison inmates, different nationalities, lay people from the community) to better understand the displayed correlations and CM effects.

Finally, lie- and truth-related ability assessments and narcissistic scores are based on self-reports. Such correlational analyses are limited in presenting real effects. In fact, self-assessed lie-truth related abilities are biased and do not necessarily reflect actual abilities. Still, as the present results show, these biased assessments may affect behavior. It was demonstrated that frequent lying in the CIT (CMs) was correlated with biased assessments of lying ability and narcissistic features.

## Conclusion

The present study provides a first look at how biased lie-truth ability assessments and narcissistic features correspond to the use of frequent CMs in the CIT. High self-assessed lie-telling ability raters frequently used CMs when they were guilty. Similar results were obtained for narcissistic features. The present results integrate into a more general concept of self-efficacy (Bandura, 1977, 1992). Following the self-efficacy model, people’s confidence in their ability to be convincing when they are truthful and be persuasive when they are lying may determine their performance in various social interactions, including a tendency to frequently (or infrequently) lie in the CIT. Future research should study additional behaviors that might be associated with high self-assessed lie-telling ability ratings. This will result in better understanding of the bonds between self-efficacy and actual lying and highlight practical implications.

## Ethics Statement

This study was carried out in accordance with the recommendations of Ariel University ethics committee guidelines with written informed consent from all subjects. The protocol was approved by the Ariel University ethic committee.

## Author Contributions

EE and LZ initiated the study, formulated the hypotheses, designed the experiments, and supervised all tasks and procedures that were conducted by research assistants. EE analyzed the data and wrote the manuscript. LZ revised and approved the manuscript.

## Conflict of Interest Statement

The authors declare that the research was conducted in the absence of any commercial or financial relationships that could be construed as a potential conflict of interest.
